# Retraction: Development of Timolol Maleate-Loaded Poloxamer-co-Poly (acrylic acid) based hydrogel for controlled drug delivery

**DOI:** 10.1371/journal.pone.0324358

**Published:** 2025-05-09

**Authors:** The PLOS One Editors

Following the publication of this article [1], concerns were raised regarding results presented in Figs 8–11. Specifically, multiple areas both within and between multiple panels presented in Figs 8–11 appear highly similar.

The corresponding author stated that the histology images presented in this article were outsourced to external laboratories, and that the images were incorporated as received. PLOS notes that the outsourcing of the histology experiments was not declared in the article, and that authors are responsible to ensure the validity, reliability, and integrity of any outsourced material included in their article.

In light of the nature and extent of these concerns that call into question the reliability and integrity of the published results, the *PLOS One* Editors retract this article.

RM and KB did not agree with the retraction. IA, MA, SFB, RU, ZI, and MAR either did not respond directly or could not be reached.
